# Examining the Effect of Awareness-Raising Efforts and Rape Myths on Attitudes Toward Survivors of Sexual Assault

**DOI:** 10.5964/sotrap.9965

**Published:** 2023-12-05

**Authors:** Melike Avcı, Ela Arı

**Affiliations:** 1 Department of Psychology, Istanbul University, Istanbul, Turkey; 2 Department of Psychology, Istanbul Medipol University, Istanbul, Turkey; Centre for Criminology (Kriminologische Zentralstelle – KrimZ), Wiesbaden, Germany

**Keywords:** survivor, rape myths, attitude, awareness video, scenario, Turkey

## Abstract

The aim of this study is to measure the effect of rape myths and an awareness raising video on attitudes toward survivors of sexual assault. Participants were exposed to rape myths presented in nontraditional, traditional, and neutral conditions, followed by an awareness video explaining these myths. Attitudes were measured both before and after the video in each scenario condition, with a total of N = 101 participants from Turkey. A 3x2 factorial design was employed, utilizing the Illinois Rape Myth Acceptance Scale (Payne et al., 1999, https://doi.org/10.1006/jrpe.1998.2238) and Attitudes toward Rape Victim Scale (Ward, 1988, https://doi.org/10.1111/j.1471-6402.1988.tb00932.x). The results indicated a positive shift in attitudes following the awareness video, with significant effects observed in the traditional and nontraditional scenario conditions but not in the neutral condition. Positive correlations were found between rape myths and attitudes toward rape victims, suggesting that an increase in rape myths was associated with more negative attitudes toward survivors. Gender differences were not observed in terms of rape myths, but females exhibited a positive change in attitudes after the awareness video, while males did not.

## Clinical Impact Statement

This study provides helpful insights for a wide range of professionals working with survivors of sexual violence, helping to understand how attitudes toward different lifestyles can change. It emphasizes the essential role of awareness-raising activities in delivering support to survivors and intervention professionals. In the context of our fast-paced society, future research should prioritize the implementation of various concise information initiatives, thereby creating opportunities for disadvantaged individuals.

## Sexual Assault

Sexual assault refers to sexual acts that occur without a person’s consent (Schulhofer, 1995). In Turkish criminal law, sexual assault is described as actions that violate a person’s bodily integrity through sexual behavior, while rape specifically involves the insertion of an organ or object into the body. In the context of this study, the term “sexual assault” was used to encompass both major forms of sexual assault and rape as defined by Turkish Criminal Law (Turkish Criminal Law, 2004).

## Awareness Raising and Prevention Programs for Sexual Assault

Sexual offending is influenced by four key factors: an increased motivation towards sexual behavior, reduced internal inhibitions such as the acceptance of rape myths, decreased external barriers related to factors like location or timing, and a reduced ability of survivors to resist including issues like physical strength (Finkelhor & Araji, 1986).

Studies on awareness-raising activities aimed at preventing sexual assault have consistently demonstrated that interventions designed to reduce the acceptance of rape myths are effective in reducing abusive behaviors as well (Adıgüzel, 2019; Breitenbecher, 2000; Foubert & Marriott, 1997; Gilbert et al., 1991; O’Donohue et al., 2003). For example, video-based education programs have been effective in preventing sexual aggression by reducing the acceptance of rape myths and increasing empathy with survivors (O’Donohue et al., 2003). Studies have shown that education programs, which have lasted between 75 minutes (O’Donohue et al., 2003) and 120 minutes (Frazier et al., 1994), produced positive changes in attitudes in the short term. In the fast-paced information age, video training has become increasingly common and efficient for enhancing learning (Brecht, 2012). In light of this contemporary trend, this research aim to investigate the impact of much shorter video training sessions lasting only 2 minutes and 21 seconds on attitudes toward rape myths.

It is expected that a significant positive change in attitudes will occur by measuring differences before and after the intervention. This study may contribute to the existing literature by addressing the impact of very brief awareness-raising activities, as no prior research on the effects of such short-term interventions has been identified to the best of the available knowledge.

## Rape Myths

Rape myths are false beliefs and stereotypes about sexual assault. While these myths are factually incorrect, they remain widely prevalent and can potentially rationalize male sexual aggression towards women (Lonsway & Fitzgerald, 1994). According to these myths, survivors are perceived as wanting or deserving the sexual assault, with only specific categories of women considered as vulnerable targets. Additionally, the myths associate aspects of a survivor’s lifestyle, such as their clothing choices or nighttime activities, as causative factors for sexual assault (Brownmiller, 1975). These myths are rooted in historical, patriarchal systems that have persisted for centuries, ultimately liberating perpetrators and blaming survivors, while influencing public perceptions of sexual assault (Brownmiller, 1975; Whatley, 1996). The acceptance of rape myths serves to minimize the culpability of the perpetrator and legitimizes sexual assaults within a putative rational framework (Payne et al., 1999).

## Attitudes Toward Survivors of Sexual Assault

The acceptance of rape myths is associated with an increased tendency to attribute responsibility to the survivor (Burt, 1980; Gray, 2006; Krahé, 1988). While earlier research suggested no gender differences in the acceptance of rape myths (Burt, 1980; Krahé, 1988), more recent studies have found that males tend to score higher than females in this regard (Grubb & Turner, 2012; Hammond et al., 2011). This difference suggests that in the last thirty years, females have become less likely to accept rape myths, while males still tend to accept them.

In alignment with male acceptance of rape myths, they tend to be more accusatory toward survivors compared to females. However, when the survivor is described without reference to gender norms or lifestyle such as alcohol consumption, participants’ inclination to assign blame decreases (Grubb & Turner, 2012). Conversely, in another study alcohol was not found to be significantly related to attributing responsibility to the survivor (Krahé, 1988). It is important to note that alcohol consumption is one of the factors included in the *Illinois Rape Myth Acceptance Scale* (Payne et al., 1999) and may still influence people’s judgments. Hence, it is important to study the relationship between alcohol consumption and lifestyle in Turkey, a country with Western influences, secular values, and Islamic traditions in the Middle East.

## The Current Study

This study aims to evaluate the influence of rape myths and awareness-raising initiatives on attitudes towards sexual assault survivors by utilizing hypothetical scenarios that reflect traditional, nontraditional, and neutral lifestyles conditions within the context of Turkish culture, augmented by video-based interventions (Çelik Ok, 2019).

While sexual assault has been explored across various disciplines in Turkey, its presence in the psychology literature is relatively scarce (Çamaş & Meşe, 2016; Çelik Ok, 2019; Çoklar & Meşe, 2014; Eker & Erdener, 2011; Yancı & Polat, 2019). This research’s unique contribution lies in its experimental design, aimed at uncovering the impact of rape myths and awareness-raising videos on attitudes. This approach could contribute to develop information materials and awareness campaigns in the public interest.

## Method

### Participants

The study recruited college students aged 18 and above offering them course credit in exchange for their participation (*M_Age_* = 22.6, *SD* = 3.58) in Istanbul, Turkey. The sample size of *N* = 101 participants exceeded the calculated minimum requirement of 42, determined through G power analysis (effect size: 0.25; alpha error: 0.05; power: 0.8; number of groups: 3; number of measurements: 2; correlation among repeated measures: 0.5; nonsphericity correction: 1). Demographic characteristics of the participants are described in Table 1.

**Table 1:** Demographic Characteristics of the Participants

Characteristic		
	*n*	%
Sex		
Female	88	87.1
Male	13	12.9
SES		
Very low	2	2.0
Low	8	7.9
Medium	64	63.4
High	27	26.7
SPWTS		
Village	3	3
Town	9	8.9
City	9	8.9
Metropolis	80	79.2
Education		
College student in 1st year	2	2.1
College student in 2nd year	35	37.2
College student in 3rd year	35	37.2
College student in 4th year	17	18.1
Master and Postgraduate	5	5.3
Romantic Relationship		
Yes	44	43.6
No	57	56.4
Harassment History		
Yes	61	60.4
No	40	39.6
Abuse History		
Yes	31	30.7
No	70	69.3
Sexual Assault History		
Yes	13	12.9
No	88	87.1
SSB		
Never	10	9.9
Rare	11	10.9
Sometimes	26	25.7
Often	43	42.6
Always	11	10.9
	*M*	*SD*
Age		
	22.13	2.38

*Note.* SES = Socioeconomic status; SPWTS = Size of the places where they spent most of their lives; SSB = Frequency of seeking support from their spiritual beliefs.

Prior to participation, all individuals provided informed consent and were ensued of the protection of their anonymity. This study was approved by Social Scientific Research Ethics Committee of the university (Ref. E-43037191-604.01.01-4435).

### Design

A 3 (traditional, non-traditional, and neutral scenarios) x 2 (awareness video watched or not watched) pretest-posttest between-groups design was employed. This design involved comparing scenario groups before and after video manipulation. Additionally, within each scenario condition, within-groups designs were used to investigate changes in attitudes before and after exposure to the awareness video. The independent variables included rape myths and awareness video, while the dependent variable consisted of attitudes toward survivors.

### Measures

#### Sociodemographic Variables

Various demographic information was gathered including age, gender, socioeconomic status, level of education, relationship status, history of harassment, abuse, and sexual assault, frequency of seeking support from spiritual beliefs, and the environments where the participants have primarily resided.

#### The Illinois Rape Myth Acceptance Scale – Short Form (IRMA-SF)

The *Illinois Rape Myth Acceptance Scale* (IRMA), developed in 1999 by Payne et al., was employed to assess rape myths. A strong correlation of .97 exists between the short form and the original version of the scale (Çoklar & Meşe, 2014). The Turkish adaptation of the short form, known as ITMS Short Form (ITMSSF) demonstrated a strong internal consistency of α = .90, item-total score correlations ranged from .34 to .65. The ITMSSF consists of 17 items, which are loading on one dimension, and are using a 7-point Likert type scale. Notably, there are no reverse-coded items within the scale, with higher scores indicating a greater endorsement of rape myths and a greater tendency to blame survivors.

#### The Attitudes Toward Rape Victims Scale

The *Attitudes toward* *Rape Victims Scale*, developed by Ward in 1988, was used to assess attitudes towards survivors. The scale was validated in Turkish by Yalçın in 2006, demonstrating strong reliability with a coefficient of .α = 90. It comprises 25 items and employs a 6-point Likert-type scale; Items 3, 5, 7, 10, 12, 15, 19, and 22 are reverse-coded. Higher scores on this scale reflect a more prejudiced and less favorable attitude towards survivors.

#### Hypothetical Event Scenarios

The scenarios are taken from a Turkish researcher Çelik Ok’s (2019) doctoral thesis, which has been tested for validity and reliability purposes with a student sample. The scenarios are divided into three conditions that included a woman with traditional lifestyle (Scenario A), a woman with nontraditional lifestyle (Scenario B), and a neutral one (Scenario C).

**Traditional Scenario Condition**

*Ayşe is employed in the human resources department of a local company. She abstains from consuming alcohol and typically opts for modest attire. Having lived with her college roommates during her academic years, Ayşe recently graduated from university and has since moved back in with her family.*

*On a particular day, Ayşe and her colleague Kemal found themselves working late hours and had to complete an overtime shift. As they wrapped up their work late at night, Kemal offered to provide Ayşe with a ride home in his car. Despite Ayşe's refusal, Kemal forcibly compelled her into his vehicle and sexually assaulted her.*

**Non-Traditional Scenario Condition**

*Ayşe is employed in the human resources department of a local company. She occasionally consumes alcohol and tends to favor revealing clothes in her attire choices. During her college years, Ayşe lived with her boyfriend, but she recently graduated from university and now resides independently.*

*On a particular day, Ayşe and her colleague Kemal found themselves working late hours and had to complete an overtime shift. As they wrapped up their work late at night, Kemal offered to provide Ayşe with a ride home in his car. Despite Ayşe's refusal, Kemal forcibly compelled her into his vehicle and sexually assaulted her.*

**Neutral Scenario Condition**

*Ayşe is employed in the human resources department of a local company.*

*On a particular day, Ayşe and her colleague Kemal found themselves working late hours and had to complete an overtime shift. As they wrapped up their work late at night, Kemal offered to provide Ayşe with a ride home in his car. Despite Ayşe's refusal, Kemal forcibly compelled her into his vehicle and sexually assaulted her.*

#### Awareness Raising Video

The awareness-raising video utilized in this study was sourced from Blue Seat Studios’ video repository^1^1https://www.youtube.com/watch?v=gNnO8VIdLzk&t=1s. The video conveyed information about sexual assault through the use of animated characters and had a duration of 2 minutes and 21 seconds. The video included English dubbing and provided additionally Turkish subtitles. The selection of an animated format aimed to prevent exposing participants to distressing content. The video mainly highlighted fallacy of rape myths.

### Data Analytic Plan

Participants were randomly assigned to one of three groups, each exposed to different scenarios: traditional, non-traditional, and neutral. These groups served as the primary categories of analysis with respect to rape myths. To assess the influence of rape myths on attitudes, a Kruskal-Wallis test was conducted to compare these three main groups. Subsequently, three separate Mann-Whitney *U* tests were performed to compare pairs of groups. To assess the impact of the video across all scenario conditions, the total attitude scores before and after viewing the video were analyzed using the Wilcoxon Signed Rank test. Furthermore, three distinct Wilcoxon Signed Rank tests were conducted to investigate differences within each scenario condition.

To explore the influence of demographic variables, subgroups were delineated for all participants based on their sex, whether they had experienced sexual abuse, sexual assault, sexual harassment, and their relationship status. Gender-specific comparisons were made between males and females, as well as between participants with or without a current romantic relationship, using the Mann-Whitney *U* test. Subsequently, separate Wilcoxon Signed Rank tests were employed to examine the effect of the video on attitude changes within females and males. For the groups categorized based on biographical experiences (sexual abuse, assault, and harassment), a Kruskal-Wallis test was used to compare these three experiences followed by three separate Mann-Whitney *U* tests were performed to compare pairs of groups. Finally, Wilcoxon Signed Rank tests were conducted to investigate the influence of the awareness video within each group.

### Procedure

First, participants completed the demographic questionnaire and the ITMSSF. Following this, they were presented with one of three scenarios—Scenario A, B, or C—and completed the *Attitude toward Rape Victims Scale*. One week later, all participants engaged in an orientation task, revisited their respective scenario (A, B, or C), watched the awareness-raising video, and completed the *Attitude toward Rape Victims Scale* once more.

## Results

The statistical analyses were performed using SPSS v.25. The groups were not normally distributed as indicated by Shapiro Wilk normality test results (*p* < .01). Prior to the intervention, attitudes exhibited a positive correlation with rape myths, *r*(100) = .36, *p* < .01. After viewing the video, a positive correlation between rape myths and post-video attitudes persisted, *r*(100) = .32, *p* < .01. Also, the frequency of seeking support from spiritual beliefs was found to have no significant correlation with attitudes (*r* = .20, *p* = .05).

### Demographics

In the present study, an analysis of gender-related differences in the acceptance of rape myths was conducted, and the findings revealed that there was no statistically significant difference between males and females in this regard (*p* = .73). Conversely, prior to exposure to an awareness video intervention, females (*Mdn* = 35.00) exhibited more favorable attitudes when compared to males (*Mdn* = 40.00) towards survivors. This difference was statistically significant, as evidenced by the Mann-Whitney *U* test, *U* (*n*_women_ = 88, *n*_men_ = 13) = 335.00, *z* = -2.40, *p* = .02). Additionally, it was observed that the presence or absence of a romantic relationship did not result in statistically significant differences in attitudes (*p* = .45).

### Pre-Awareness Video Context

A significant overall difference was observed among the main groups (traditional, non-traditional, and neutral scenario conditions) in terms of attitudes influenced by rape myths, *H*(2) = 7.39, *p* = .03. Subsequent pairwise comparisons indicated no significant differences between the traditional and non-traditional groups (*p* = .37), as well as between the non-traditional and neutral groups (*p* = .11). However, the neutral scenario group (*Mdn* = 33.00) displayed more favorable attitudes compared to the traditional group, *Mdn* = 39.00, *U* (*n*_traditional_ = 32, *n*_neutral_ = 32) = 301.00, *z* = -2.84, *p* = .01.

Before exposure to the awareness video, there were no discernible differences in attitudes between survivors and non-sexually assaulted participants (*p* = .55). Similarly, no significant differences in attitudes were observed between sexually harassed and non-sexually harassed groups prior to the intervention (*p* = .67). However, a distinction emerged between the sexually abused group (*Mdn* = 34.00) and the non-sexually abused group (*Mdn* = 38.00) concerning attitudes before the intervention, as evidenced by the Mann-Whitney *U* test, *U* (*n*_abused_ = 31.00, *n*_not abused_ = 37.50, *z* = -2.56, *p* = .01).

### Effect of the Awareness Video on Attitudes

The total scores of attitudes exhibited a statistically significant positive change after exposure to the awareness video (*Mdn* = 32.00) compared to pre-intervention attitudes (*Mdn* = 35) for all participants, *t* = 625, *z* = -5.37, *p* = .001. Upon closer examination of the individual scenario groups, the awareness video effectively reduced negative attitudes among participants exposed to the traditional scenario, both before (*Mdn* = 39.50) and after (*Mdn* = 33.50) intervention, *t* = 35.50, *z* = -3.27, *p* = .001. Similarly, the awareness video led to a significant reduction in unfavorable attitudes within the non-traditional scenario group, both before (*Mdn* = 35) and after (*Mdn* = 32) the intervention, *t* = 60.00, *z* = -3.82, *p* = .001. However, it is noteworthy that the awareness video did not result in a significant change in attitudes among participants exposed to the neutral scenario (*p* = .06).

After the intervention involving the awareness-raising video, it was observed that men (*Mdn* = 36.00) obtained significantly higher attitude scores toward survivors compared to women (*Mdn* = 32.00), *U* (*n*_women_ = 88, *n*_men_ = 13) = 299.50, *z* = -2.79, *p* = .001. Consequently, it is noteworthy that women exhibited a statistically significant positive change in their attitudes towards survivors after the video intervention, *t* = 407.50, *z* = -5.29, *p* < .01, whereas men did not exhibit a significant change in their attitudes toward survivors (*p* = .21).

Participants who had not experienced sexual assault demonstrated a statistically significant positive change in their attitudes before (*Mdn* = 35.00) and after (*Mdn* = 32.00) the intervention (*t* = 359.50, *z* = -5.54, *p* = .001). Conversely, survivors of sexual assault did not exhibit a significant change in their attitudes following the video manipulation (*p* = .85). The sexually harassed and non-sexually harassed groups displayed similar levels of attitudes after the intervention (*p* = .68). Notably, the attitudes of the sexually harassed group exhibited a positive change from before (*Mdn* = 35.00) to after (*Mdn* = 32.00) the video manipulation, *t* = 254.50, *z* = -3.83, *p* = .001. These findings indicate that also the sexually harassed participants were receptive to the informative video and showed a favorable shift in their attitudes following the intervention.

A significant difference was found between the sexually abused group (*Mdn* = 31.00) and the not sexually abused group (*Mdn* = 33.50) in terms of attitudes after the intervention video, with the sexually abused group displaying more favorable attitudes, *U* (*n*_abused_ = 31.00, *n*_not abused_ = 37.00, *z* = -2.85, *p* = .001). Furthermore, it was observed that the attitudes of the sexually abused group decreased from before (*Mdn* = 34.00) to after (*Mdn* = 31.00) watching the video, indicating a significant positive change, *t* = 55.50, *z* = -3.37, *p* = .001. These results suggest that the intervention video had a beneficial impact on the attitudes of the sexually abused group, leading to more favorable attitudes following the intervention.

## Discussion

This study aimed to investigate the effect of rape myths on attitudes toward survivors. As anticipated, a strong connection was found between rape myths and attitudes toward survivors. Even though all participants shared similar levels of belief in rape myths, exposure to different scenarios involving these myths led to varied attitudes. Specifically, participants exposed to the traditional scenario displayed more negative attitudes toward survivors compared to those exposed to the neutral scenario. This alignment with traditional gender roles, which are positively associated with the acceptance of rape myths (Anderson & Cummings, 1993; Cooke et al., 2022), appears to contribute to the development of these negative attitudes. Therefore, negative attitudes toward survivors seem to result from their association with rape, regardless of norm violations.

Rather than focusing solely on survivors’ lifestyles, it is crucial to examine the social perceptions of rape cases and victim blaming through the lens of observer characteristics (Acock & Ireland, 1983; Scurlock-Evans, 2019). Participants in the non-traditional scenario condition, with lower belief in rape myths, appeared to identify more closely with the characters, possibly due to ingroup bias (Mackie & Smith, 1998), resulting in more positive attitudes. Conversely, the more negative attitudes toward the traditional scenario group, perceived as dissimilar, can be explained by out-group derogation (Hewstone et al., 2002). The second aim of this study was to assess the impact of a brief awareness-raising video on attitudes toward survivors. The results demonstrated the effectiveness of the video in raising positive attitude changes, particularly in the traditional and non-traditional scenario conditions. However, this significant effect was not observed in the neutral scenario condition. Interestingly, despite the influence of rape myths on participants’ guilt-related decisions, the neutral scenario did not differ significantly from other scenarios, including those supporting or opposing rape myths (Gray, 2006).

This study revealed that both traditional and non-traditional scenarios had a similarly positive impact on attitudes, regardless of the manipulated survivor’s lifestyle. This aligns with previous research indicating that a survivor’s adherence to traditional roles or their survivor type did not influence the likelihood of sexual assault (Masser et al., 2006). Notably, participants did not exhibit a negative approach toward the survivor's lifestyle, possibly due to their low scores on rape myth beliefs. These findings are consistent with previous research that found a low acceptance of rape myths (Hayes-Smith & Levett, 2010), and since believing in such myths is linked to negative attitudes toward survivors (Burt, 1980; Gray, 2006; Krahé, 1988), participants with low myth scores may have experienced similar attitude changes in both non-traditional and traditional conditions.

The participants’ low belief in rape myths may be associated with the demographics of the sample, consisting of undergraduate psychology students. Psychologists are typically trained to be unbiased, which might explain their reduced inclination to endorse rape myths compared to individuals in other professions. Previous research has also indicated that psychologists tend to assign less blame to survivors compared to those in other professions (Gölge, 1997). These factors may have contributed to the consistent attitude changes observed across scenario conditions in this study.

In this study, rape myth scores did not show significant differences between male and female participants. This finding differs from some prior research where males tended to score higher in believing rape myths compared to females (Grubb & Turner, 2012; Hammond et al., 2011; Hayes-Smith & Levett, 2010; Örüklü et al., 2021). However, it aligns with other studies that found no significant differences in rape myth scores between males and females (Burt, 1980; Krahé, 1988). One plausible explanation for this discrepancy might be the relatively low number of male participants in this study, which may have limited the statistical power to detect differences. Additionally, the similarity in educational backgrounds between male and female participants could have contributed to the lack of divergence in their rape myth scores. Further research with a larger and more diverse sample can provide a more comprehensive insight into gender differences in beliefs regarding rape myths. However, the results of the current study revealed that men demonstrated more negative and less favorable attitudes toward survivors compared to women. This finding aligns with prior research that has consistently shown that men tend to hold more negative attitudes toward survivors and engage in victim-blaming behaviors (Davies et al., 2012; Gray, 2006; Johnson et al., 1997).

Being a survivor of sexual assault emerged as a significant factor associated with attitude change in this study. Survivors of sexual assault displayed greater resistance to positive attitude changes compared to participants who had not experienced such trauma. This finding suggests that survivors may exhibit resistance to the effects of awareness videos and attitude change interventions. This study results align with prior research, indicating that contemplating a rape scenario may reduce sympathy for survivors and may increase victim-blaming tendencies (Ellis et al., 1992). However, it is important to recognize that survivors’ reactions and attitudes can vary widely depending on multiple factors, including age, life situation, beliefs in rape myths, the response of the justice system, family support, and the support of friends. Additionally, emotions such as guilt, shame, self-blame, and feelings of loss of control play crucial roles in shaping survivors’ attitudes toward rape (Hendricks & Hendricks, 2014). These factors underscore the complexity of survivors’ responses and highlight the need for a comprehensive approach to support and intervention.

On the contrary, participants who had experienced sexual harassment showed similar attitude changes to those who had not experienced it after viewing the awareness video. Both groups were receptive to the informative video, indicating an absence of resistance to its effects. However, participants who had experienced sexual assault did not show significant changes in their attitudes after the awareness video. Interestingly, sexually abused participants did show positive attitude changes following the intervention. Especially, sexually abused participants differed from their non-sexually abused counterparts both before and after the intervention, displaying more positive attitudes. The distinction between these groups may be attributed to the broader psychological and emotional consequences associated with being a survivor of sexual abuse, including higher rates of substance abuse, binge eating, somatization, suicidal behaviors, poorer social and interpersonal relationships, greater sexual dissatisfaction and dysfunction, and an increased susceptibility to revictimization through adult sexual assault and physical partner violence (Polusny & Follette, 1995). However, it is worth noting that research on the impact of awareness-raising activities on sexually abused individuals remains scarce. Additionally, current study’s sexually abused group primarily comprised women, with only one male participant. Research has indicated that women tend to exhibit more prosocial beliefs, attitudes, and emotional reactions toward issues related to sexual abuse, abusers, and victims when compared to men (Kamas & Preston, 2021; Wellman, 1993). This increased sensitivity among women may have contributed to their positive response to the awareness-raising video.

A positive correlation was observed between belief in myths and participants’ attitudes, confirming the expectations of this study. Specifically, as belief in myths increased, participants exhibited more negative attitudes towards survivors. This finding aligns with prior research (Brownmiller, 1975; Payne et al., 1999). Similar results were reported in studies conducted in Turkey (Yancı & Polat, 2019), reinforcing the stable relationship between rape myths and unfavorable attitudes (Brownmiller, 1975; Burt, 1980; Krahé, 1988). This correlation was once again evident in this study among college students in Turkey.

### Limitations

The low myth scores among participants posed challenges in assessing the impact of myths on attitudes. Diverse groups with varying levels of acceptance of rape myths may have provided more comprehensive insights into the effects of these myths in scenarios. Additionally, a limitation of this study was the limited number of male participants, which diminished the generalizability of the findings. In order to determine whether there is a gender difference in terms of rape myth acceptance of, it is recommended to conduct further research involving a more extensive and diverse sample. Furthermore, the impact of the awareness-raising video was assessed primarily among female participants. Future studies should aim to include a larger proportion of male participants in order to provide a more balanced perspective. Given that perpetrators often target individuals they perceive as vulnerable, it remains crucial to implement educational programs aimed at protecting women. However, it is equally important to educate men about recognizing and responding to sexual assault, as emphasized by Brownmiller (1975). This inclusive approach can contribute significantly to addressing the issue effectively.

In this current study, the impact of traditionalism, which has been shown to have a negative influence on attitudes towards survivors was not explored (Acock & Ireland, 1983). Additionally, data on demographic variables and rape myth acceptance was collected but did not include measures of self-blame, guilt, or shame. These variables should be considered for investigation in future studies, as they may provide valuable insights (Hendricks & Hendricks, 2014).

### Conclusion

The study’s findings showed that an awareness-raising video could have a positive effect on participants, irrespective of their pre-existing beliefs in rape myths. Survivors of sexual assault displayed resistance to the awareness-raising activity and did not exhibit a positive change in their attitudes, whereas participants who had experienced sexual abuse or harassment did show positive change in their attitudes. Additionally, it was observed that men held more negative and unfavorable attitudes toward survivors compared to women.

## Data Availability

The data that support the findings of this study are available from the corresponding author upon reasonable request.
